# Gender Difference in the Relationship between Extrapulmonary Factors and Reduced Lung Function in Early Adulthood

**DOI:** 10.3390/jcm13061769

**Published:** 2024-03-19

**Authors:** Keiko Doi, Tsunahiko Hirano, Keiji Oishi, Ayumi Fukatsu-Chikumoto, Yuichi Ohteru, Kazuki Hamada, Shuichiro Ohata, Yoriyuki Murata, Yoshikazu Yamaji, Maki Asami-Noyama, Nobutaka Edakuni, Tomoyuki Kakugawa, Kazuto Matsunaga

**Affiliations:** 1 Department of Respiratory Medicine and Infectious Disease, Graduate School of Medicine, Yamaguchi University, 1-1-1 Minami-kogushi, Ube, Yamaguchi 755-8505, Japan; decem119@yamaguchi-u.ac.jp (K.D.); teru1153@yahoo.co.jp (Y.O.); khamada@yamaguchi-u.ac.jp (K.H.); edakuni@yamaguchi-u.ac.jp (N.E.); kazmatsu@yamaguchi-u.ac.jp (K.M.); 2 Department of Pulmonology and Gerontology, Graduate School of Medicine, Yamaguchi University, 1-1-1 Minami-kogushi, Ube, Yamaguchi 755-8505, Japan; kakugawa@yamaguchi-u.ac.jp

**Keywords:** gender difference, early adulthood, extrapulmonary factors, impaired lung function, body composition, handgrip strength

## Abstract

(1) **Background**: Reduced lung function in early adulthood is associated with future risks to health outcomes that have not been fully explored by gender. We investigated gender-specific relationships between lung function and extrapulmonary variables, assessing their potential as screening markers for respiratory dysfunction in young adults. (2) **Methods**: The participants were 151 medical students. Clinical data, handgrip strength (HS); body composition parameters such as skeletal muscle mass index (SMI), whole-body phase angle (WBPhA), and bone mineral content (BMC); and pulmonary function variables, vital capacity (VC), forced VC (FVC), and forced expiratory volume in one second (FEV_1_), were measured. (3) **Results**: FEV_1_ was significantly correlated with BMI, SMI, WBPhA, BMC, and both left and right HS (*p <* 0.0001, respectively) across all participants. According to gender, FEV_1_ had the strongest positive association with left HS in males (*p <* 0.0001) and BMC in females (*p <* 0.0001). The area under the curve for detecting the bottom quartile of FEV_1_ was 0.705 (cut-off 41.0 kg, sensitivity 91%) for left HS in males and 0.742 (cut-off 2.11 kg, sensitivity 81%) for BMC in females. (4) **Conclusions**: Gender-specific relationships between intrapulmonary and extrapulmonary factors such as left HS and BMC could be useful for screening suspected respiratory dysfunction in early adulthood.

## 1. Introduction

Reduced lung function, which can be caused by obstructive and restrictive diseases and bronchopulmonary hypoplasia, etc., is associated with mortality, cardiovascular disease, and respiratory events [1]. For example, it has been reported that reduced lung function in early adulthood is associated with heart failure, arrhythmia, stroke, COPD, and obesity in the future [2,3,4,5]. Among them, COPD prevalence has been growing worldwide, an COPD can lead to physical inactivity, frailty, cognition issues, and mortality [6,7,8,9]. In addition to therapeutic drugs, approaches tailored to the characteristics of comorbidities, such as bronchiectasis, asthma, heart failure, sleep apnea, malnutrition, and frailty, have been shown to be viable in the management of COPD [10]. However, a curative treatment does not currently exist. [11]. Therefore, at this time, the practical target is the prevention of pathophysiology, for example, smoking cessation.

In the past, a progressive decline in lung function due to toxic particle inhalation by smoking or air pollution has been believed to be the hallmark of COPD. However, recently, impaired lung function in early life is also reported to be a cause of COPD [12]. This type of patient is at risk of developing COPD regardless of the rapid decline in lung function over time.

Lung function in early adulthood has been reported to be associated not only with intrapulmonary factors such as childhood asthma and pneumonia but also with extrapulmonary factors such as body composition (weight, muscle, and fat mass, etc.) and handgrip strength (HS) [13,14,15,16]. Especially in the relationship between extrapulmonary factors and lung function, it has been shown that it is important to consider gender differences due to the distinctive features of each gender’s physique [16]. Muscle mass and muscle strength are typical extrapulmonary factors, and the characteristics of the abdominal and lumbar muscles are already different during adolescence [17]. However, to date, there is little evidence on which gender-specific factors can precisely detect reduced lung function in early adulthood. If this becomes clear, it could be a valuable screening tool.

Therefore, we aimed to investigate the underlying mechanism and treatment target of the phenotype by gender and the possibility of screening for the detection of reduced lung function in early adulthood [18,19,20].

## 2. Materials and Methods

### 2.1. Study Participants

This study was a retrospective cross-sectional analysis of young adults. We recruited 151 medical students from Yamaguchi University aged between 22 and 24 years. We excluded participants with disorders that would prevent them from completing the study assessments. The study protocol and amendments were approved by the ethics committee of Yamaguchi Medical University (institutional review board no. H2019-138). Our study complied with Japanese Ethical Guidelines for Medical and Health Research Involving Human Subjects, which do not require informed consent from participants enrolled in studies not using human biological specimens. All participants, however, were given the option to opt out of the study.

### 2.2. Study Assessments

According to the recommendations of the American Thoracic Society and European Respiratory Society [21], pulmonary function was assessed using a multifunctional spirometer, the HI-801 (Chest Ltd., Tokyo, Japan). The Japanese reference values for pulmonary function were used [22]. Handgrip strength (HS) was measured using a Digital Grip Dynamometer (Takei Scientific Instruments Co., Ltd., Niigata, Japan) as follows. The participants were instructed to stand upright, let their arm hang naturally, and clasp the grip with full force. Two readings were obtained from the right and left hands, and the higher score was used as the measured HS value for each [23]. Body composition parameters such as skeletal muscle mass index (SMI), whole-body phase angle (WBPhA), fat mass, and bone mineral content (BMC) were measured by bioelectrical impedance analysis using the InBody S10 (InBody Japan Inc., Tokyo, Japan) while a participant was in the supine position [24]. Birth weight, disease, and smoking history were assessed using self-administered questionnaires.

### 2.3. Statistical Analyses

Continuous variables are shown as the median (interquartile range), and categorical variables are presented as numbers. Differences between continuous variables were assessed using the Wilcoxon test. Categorical data were compared using Pearson’s chi-squared test. Spearman’s rank correlation and multiple linear regression analyses using the least squares method were performed to analyze the correlations between the parameters. We analyzed the sensitivity and specificity using the area under the curve (AUC) to identify a cut-off value for the clinical variables to detect the bottom quartile for lung function. Statistical analyses were performed using JMP Pro^®^, version 16.1.0 (SAS Institute, Inc., Cary, NC, USA). Statistical significance was set at a probability (*p*) value of <0.05.

## 3. Results

The characteristics of the study participants are listed in Table 1. In total, 86 males and 65 females with a median age of 23.0 years participated in the study. Eleven participants had a history of smoking. Overall, 23, 24, and 68 participants had a history of asthma, pneumonia, and allergic rhinitis, respectively. Body composition parameters, such as height, BMI, skeletal muscle mass index (SMI), whole-body phase angle (WBPhA), and bone mineral content (BMC), were significantly higher in the males than in the females (all *p <* 0.0001). The right and left handgrip strength (HS) values for the males were significantly higher than those for the females (44.2 vs. 25.8 kg and 40.0 vs. 23.4 kg, respectively; *p <* 0.0001). The absolute values of lung function, vital capacity (VC), forced vital capacity (FVC), and forced expiratory volume in one second (FEV_1_) were significantly higher in the males than in the females (all *p <* 0.0001). However, FEV_1_/FVC (%) did not differ between the genders. In addition, FEV_1_/FVC < the lower limit of normal (LLN) (%) was significantly higher in the females than in the males (*p* = 0.0389).

Table 2 shows the correlations between extrapulmonary factors and lung function in all participants. FVC was significantly correlated with BMI (*p <* 0.0001), SMI (*p <* 0.0001), WBPhA (*p <* 0.0001), BMC (*p <* 0.0001), right and left HS (*p <* 0.0001), and birth weight (*p* = 0.0083). FEV_1_ was also significantly correlated with BMI (*p <* 0.0001), SMI (*p <* 0.0001), WBPhA (*p <* 0.0001), BMC (*p <* 0.0001), right and left HS (*p <* 0.0001), and birth weight (*p* = 0.0043). However, fat mass was not significantly correlated with lung function. VC also showed similar results (Table S1: Univariate correlation analysis of extrapulmonary factors for VC in all participants).

**Figure 1:**
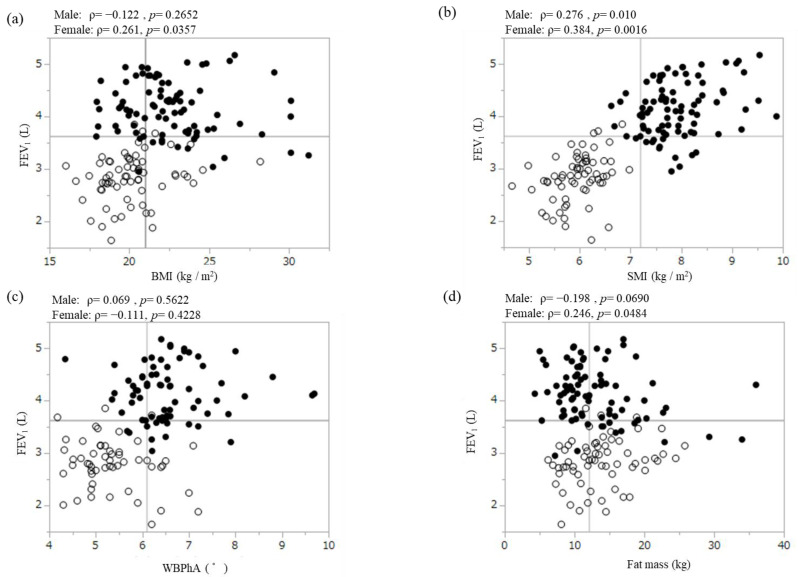

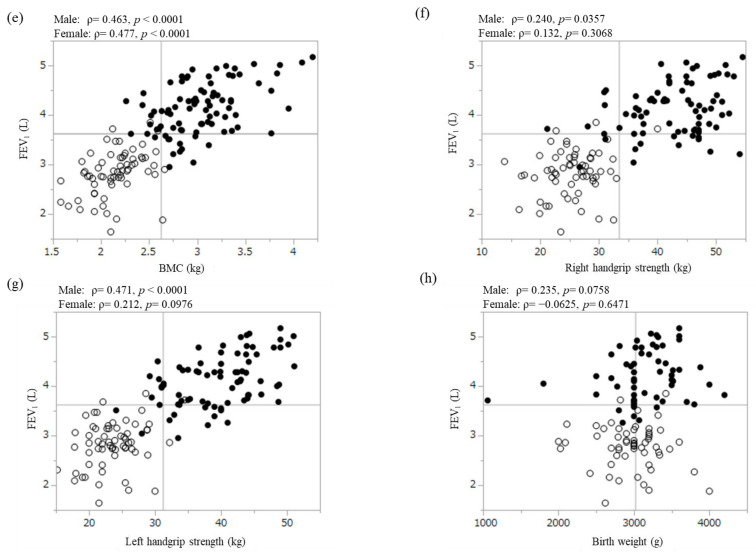
Correlation between FEV_1_ and BMI (**a**), SMI (**b**), WBPhA (**c**), fat mass (**d**), BMC (**e**), right HS (**f**), left HS (**g**), and birth weight (**h**) in all participants. The horizontal line indicates the median FEV_1_ value (3.62 L). The vertical line indicates the median BMI (21.0 kg/m^2^), SMI (7.2 kg/m^2^), WBPhA (6.1°), fat mass (12.1 kg), BMC (2.63 kg), right HS (33.5 kg), left HS (31.3 kg), and birth weight (3020 g). BMI, body mass index; SMI, skeletal muscle mass index; WBPhA, whole-body phase angle; BMC, bone mineral content; HS, handgrip strength; ●, male; ○, female.

**Figure 2:**
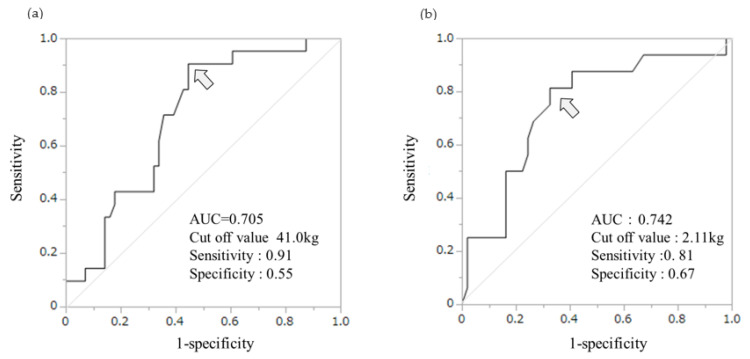
Diagnostic ability of the left HS of males (**a**) and BMC of females (**b**) to identify the bottom quartile of FEV_1_ values. The arrow indicates the cut-off point (41.0 kg) for the left HS and BMC (2.11 kg). HS, handgrip strength; BMC, bone mineral content; AUC, area under the curve.

We revealed the relationships between FEV_1_ and extrapulmonary factors (Figure 1). There was a gender difference trend in these relationships. Except for fat mass and birth weight, the males had higher values than the females in extrapulmonary factors, with the values mostly being higher than the median. Table 3 shows the correlation between the extrapulmonary factors and FEV_1_ in order of strength of correlation by gender. In the males, the strongest correlation with FEV_1_ (ρ = 0.47, *p <* 0.0001; Table 3, Figure 1g) was found in the left HS. Except for left HS, BMC (ρ = 0.46, *p <* 0.0001; Table 3, Figure 1e), SMI (ρ = 0.28, *p* = 0.010; Table 3, Figure 1b), and right HS (ρ = 0.24, *p* = 0.036; Table 3, Figure 1f) were significantly correlated with FEV_1_ in order of decreasing strength of correlation. As shown in Figure 2a, a left HS value of < 41.0 kg had 91% sensitivity and 55% specificity (AUC, 0.705) in detecting the bottom quartile of FEV_1_ (low FEV_1_). The females had the strongest correlation between BMC and FEV_1_ (ρ = 0.477, *p <* 0.0001; Table 3 and Figure 1e). Following BMC, SMI (ρ = 0.39, *p* = 0.002; Table 3, Figure 1b), BMI (ρ = 0.26, *p* = 0.036; Table 3, Figure 1a), and fat mass (ρ = 0.25, *p* = 0.048; Table 3, Figure 1d) were significantly correlated with FEV_1_ (in order of decreasing strength of correlation). A BMC value of < 2.11 kg had the highest sensitivity (81%), specificity (67%), and AUC (0.742) for detecting low FEV_1_ (Figure 2b). The trends in the strength of the correlation between VC, FVC, and the extrapulmonary factors were nearly identical across each gender (Table S2: Correlation analysis of extrapulmonary factors for VC and FVC by gender).

## 4. Discussion

Our findings showed that left handgrip strength (HS) in males and bone mineral content (BMC) in females exhibited a significantly positive association with forced expiratory volume in one second (FEV_1_). Moreover, the decline in these gender-specific extrapulmonary factors may serve as a potential screening tool for the early detection of low FEV_1_ in early adulthood.

Both airflow (measured by FEV_1_) and lung volume (measured by FVC) are positively correlated with HS in healthy young adults [14,25]. Consistent with these findings, FVC and FEV_1_ were significantly associated with HS in this study (Table 2).

In addition, this study found that there were gender differences in the correlation between HS and lung function. FEV_1_ had a strongest correlation with left HS in the males and no significant correlation with HS in the females. We found that muscle mass and strength, BMC in males, and body composition (BMC, SMI, BMI, fat mass) in females contributed to FEV_1_ in young, healthy adults. The reason for the gender difference in the relationship between lung function and extrapulmonary factors may be the divergence of ordinary breathing. Males breathe mainly through the diaphragm [26,27]. As the diaphragm is the most important respiratory muscle [28], the left and right HS, which indicate general muscle strength, may be more correlated with FEV_1_ in males than in females [16,29]. Supporting this, Park et al. reported a strong correlation between respiratory muscle strength and handgrip strength, with males showing a stronger correlation [30]. In addition, left HS, which is usually used less than right HS in daily life activities, may reflect respiratory muscle strength, including that of the diaphragm. By contrast, females breathe primarily through thoracic cavity motion [26,27]. Because their ribs are more inclined than those of males, they can make the intercostal muscles raise the ribs more efficiently [27], which results in less practice of the diaphragm than in males. Therefore, healthy rib cage mobility and thoracic volume growth may be more important for thoracic breathing. Body composition, which includes BMC, muscle, and fat mass, could show lung conditions such as healthy ribcage mobility and thoracic volume growth in healthy young females.

In fact, many studies have reported the relationship between bone mineral density (BMD) and COPD [31,32,33]. Zhang et al. showed that BMD was significantly related to FEV_1_, FVC, and disease severity in patients with COPD [31]. However, consistent findings have not reported an association between BMD and lung function in the general healthy population, and there are few reports in young healthy people. Cvijetić et al. reported that there was no correlation between lung function (FVC%, FEV_1_%, FEF50%) and bone density [34]. Possible reasons for this discrepancy include the fact that they used a prediction formula for pulmonary function and the fact that their participants were younger (19.1 ± 1.0 for boys and 19.2 ± 1.3 for girls). Although an analysis using percent predicted values was also performed in this study (Table S3: Univariate correlation analysis of extrapulmonary factors for lung function in all participants), the absolute values better reflected the relationship between extrapulmonary factors and lung functions. Utilizing predicted values may underestimate the impact of extrapulmonary factors, including physical differences. Therefore, we analyzed the absolute values. Zeng et al. reported that a reduction in BMD is associated with lower FEV_1_ and FVC in generally healthy Chinese adults from rural areas aged 40–70 years old [35]. Although we measured BMC, not BMD, in line with this result, FVC and FEV_1_ also showed a high correlation with BMC in this study. This suggests that BMC may play a role in the observed decline in lung function and could potentially be used to detect preclinical COPD, even in young and healthy individuals. BMC is important for both genders, but muscle strength appears to be equally important only in males.

While a left HS value of < 41.0 kg in males had 91% sensitivity and 55% specificity as an indicator for identifying reduced lung volume AUC (0.71), a BMC value of < 2.11 kg in females had 81% sensitivity and 67% specificity AUC (0.74). Therefore, these procedures have the potential to be used to identify the bottom quartile of FEV_1_. Actually, these cut-off values could identify FEV_1_ < the lower limit of normal (LLN) in students (six out of six males and seven out of nine females). However, both the left HS and BMC cut-off values had low specificity. This was attributed to the characteristics of the participant sample, such as its robustness, narrow age range, and small sample size. Therefore, a wider age range and a larger sample size, along with questionnaires on exercise habits, could increase their specificity as a screening tool.

This study has some limitations. Our study lacks follow-up data. The participants were still young, and follow-up is difficult. A longitudinal study is required to confirm the relationship between extrapulmonary factors and reduced lung function in future studies. The small sample size and ethnic homogeneity of the population are also limitations of this study. The percentage of LLN was higher than 5% in both males and females for all lung functions (VC, FVC, FEV_1_, FEV_1_/FVC) (Table 1), indicating that this population includes a higher than usual percentage of individuals with low lung function. This was because we recruited participants from a single university in Japan. A larger sample size and diverse young population need to be studied. Most of the participants were right-handed (102 of the 110 who answered the questionnaire), and we could not analyze the relationship between the dominant or non-dominant HS and respiratory function sufficiently. We collected data from students from a single medical university. Since they had medical knowledge, they had a lower prevalence of obesity (14 of 151), and only a few of them had a smoking history (11 of 130).

In future research, overcoming the above limitations and comparing parameters between groups of participants without underlying lung conditions and those with underlying lung conditions may confirm the results of this study.

## 5. Conclusions

The gender-specific relationship between extrapulmonary factors and expiratory airflow in young, healthy individuals is a significant finding. Notably, left handgrip strength (HS) in males and bone mineral content (BMC) in females emerge as key indicators. This distinction could be pivotal in early screening for respiratory dysfunction. Moreover, recognizing these factors may encourage young adults who are at risk of developing Chronic Obstructive Pulmonary Disease (COPD) to proactively seek lung function testing. This could be crucial for early intervention and the management of potential respiratory diseases.

## Figures and Tables

**Table 1:** Characteristics of the study participants.

Characteristics	All (*n* = 151)	Males (*n* = 86)	Females (*n* = 65)	*p*-Value
Age (years)	23.0 (22–24)	23.0 (22–24)	23.0 (22–24)	0.265
Height (cm)	167 (158–173)	172 (168–176)	157 (153–161)	<0.0001
BMI (kg/m^2^)	21.0 (19.7–23.0)	22.1 (20.6–23.9)	20.0 (18.7–20.9)	<0.0001
SMI (kg/m^2^)	7.2 (6.1–7.9)	7.77 (7.45–8.25)	5.97 (5.65–6.27)	<0.0001
WBPhA (°), *n* = 127	6.1 (5.3–6.5)	6.43 (6.02–6.95)	5.2 (4.9–5.9)	<0.0001
Fat mass (kg), *n* = 150	12.1 (9.6–15.8)	11.2 (9.6–15.2)	13 (9.8–16.3)	0.163
BMC (kg)	2.63 (2.20–3.07)	2.99 (2.74–3.28)	2.15 (1.95–2.3)	<0.0001
Rt HS (kg), *n* = 139	33.5 (26–45)	44.2 (37.4–47.1)	25.8 (22.6–28.7)	<0.0001
Lt HS (kg), *n* = 139	31.3 (23.9–41.0)	40.0 (34.1–44.0)	23.4 (21.2–25.9)	<0.0001
Birth weight (g), *n* = 114	3020 (2818–3320)	3100 (3000–3440)	3000 (2777–3200)	0.0086
Disease history				
Asthma	23	14	9	0.680
Pneumonia	24	12	12	0.428
Allergic rhinitis	68	36	32	0.402
Smoking history [Cu/Ex/Non]	5/6/139	5/5/75	0/1/64	0.0504
VC (L)	4.05 (3.16–4.73)	4.69 (4.32–5.12)	3.12 (2.9–3.34)	<0.0001
FVC (L)	4.17 (3.25–4.72)	4.63 (4.32–5.16)	3.15 (2.89–3.46)	<0.0001
FEV_1_ (L)	3.62 (2.9–4.22)	4.13 (3.72–4.49)	2.86 (2.64–3.14)	<0.0001
FEV_1/_FVC (%)	89.3 (85.1–93.4)	89.1 (83.9–92.3)	90.9 (87.1–94.5)	0.0509
%VC (%)	95.5 (87.9–103.4)	96.4 (89.7–104.7)	93.3 (86.5–102.9)	0.160
% FVC (%)	97.1 (89.3–104.9)	97.6 (90.5–106.0)	96.8 (88.2–104.3)	0.399
%FEV_1_ (%)	98.1 (90.9–105.9)	99.0 (90.9–106.5)	97.1 (90.7–104.6)	0.456
VC< LLN, *n* (%)	19 (12.6)	8 (9.3)	11 (16.9)	0.162
FVC< LLN, *n* (%)	15 (9.9)	8 (9.3)	7 (10.8)	0.765
FEV_1_ < LLN, *n* (%)	15 (9.9)	6 (7.0)	9 (13.8)	0.162
FEV_1/_FVC< LLN, *n* (%)	11 (7.3)	3 (3.5)	8 (12.3)	0.0389

BMI, body mass index; SMI, skeletal muscle mass index; WBPhA, whole-body phase angle; BMC, bone mineral content; Rt, right; Lt, left; HS, handgrip strength; Cu, current smoker; Ex, ex-smoker; Non, non-smoker; %, predicted; VC, vital capacity; FVC, forced vital capacity; FEV_1_, forced expiratory volume in one second. LLN, the lower limit of normal (LMS2014). Data are presented as medians (interquartile range) unless otherwise stated. Differences between groups were assessed using the Wilcoxon test. Categorical data were compared using Pearson’s chi-squared test.

**Table 2:** Univariate correlation analysis of extrapulmonary factors for lung function in all participants.

	FVC		FEV_1_	
	ρ	*p*-Value	ρ	*p*-Value
BMI	0.447	<0.0001	0.389	<0.0001
SMI	0.828	<0.0001	0.788	<0.0001
WBPhA	0.523	<0.0001	0.498	<0.0001
Fat mass	−0.07	0.4148	−0.11	0.1647
BMC	0.840	<0.0001	0.818	<0.0001
Rt HS	0.756	<0.0001	0.740	<0.0001
Lt HS	0.834	<0.0001	0.807	<0.0001
Birth weight	0.246	0.0083	0.265	0.0043

FVC, forced vital capacity; FEV_1_, forced expiratory volume in one second; BMI, body mass index; SMI, skeletal muscle mass index; WBPhA, whole-body phase angle; BMC, bone mineral content; Rt, right; HS, handgrip strength; Lt, left. ρ is the Spearman’s rank correlation coefficient.

**Table 3:** Correlation analysis of extrapulmonary factors for FEV_1_.

	Males			Females	
	ρ	*p*-Value		ρ	*p*-Value
Lt HS	0.471	<0.0001	BMC	0.477	<0.0001
BMC	0.463	<0.0001	SMI	0.384	0.002
SMI	0.276	0.010	BMI	0.261	0.036
Rt HS	0.240	0.036	Fat mass	0.246	0.048
Birth weight	0.235	0.076	Lt HS	0.212	0.098
WBPhA	0.069	0.562	Rt HS	0.132	0.307
Fat mass	−0.198	0.069	WBPhA	−0.111	0.423
BMI	−0.122	0.265	Birth weight	−0.062	0.647

FEV_1_, forced expiratory volume in one second; BMI, body mass index; SMI, skeletal muscle mass index; WBPhA, whole-body phase angle; BMC, bone mineral content; Rt, right; HS, handgrip strength; Lt, left. ρ is the Spearman’s rank correlation coefficient.

## Data Availability

The data analyzed during the current study are included in this article. Additional data are available from the corresponding author upon request.

## Supplementary Materials

The following supporting information can be downloaded at: https://www.mdpi.com/article/10.3390/jcm13061769/s1, Table S1: Univariate correlation analysis of extrapulmonary factors for VC in all participants. Table S2: Correlation analysis of extrapulmonary factors for VC and FVC by gender. Table S3: Univariate correlation analysis of extrapulmonary factors for lung function in all participants.
