# Supplementary material for: Gender Difference in the Relationship between Extrapulmonary Factors and Reduced Lung Function in Early Adulthood
Source: J Clin Med. 2024 Mar 19;13(6):1769. doi: 10.3390/jcm13061769 (PMC10970829; doi:10.3390/jcm13061769)
Supplement: Supplementary file 1 [file jcm-13-01769-s001.zip › jcm-2875604-supplementary.pdf]

Table S1. Univariate correlation analysis of extrapulmonary factors for VC in all participants

	VC	
	ρ	*p*-value
BMI	0.455	<.0001
SMI	0.831	<.0001
WBPhA	0.543	<.0001
Fat mass	-0.05	0.5330
BMC	0.845	<.0001
Rt HS	0.773	<.0001
Lt HS	0.840	<.0001
Birth weight	0.256	0.0060

VC, vital capacity; BMI, body mass index; SMI, skeletal muscle mass index; WBPhA, whole-body phase angle; BMC, bone mineral content; Rt, right; HS, handgrip strength; Lt, left.

ρ is the Spearman's rank correlation coefficient.

Table S2. Correlation analysis of extrapulmonary factors for VC and FVC by gender

	Males				Females			
	VC		FVC		VC		FVC	
	ρ	p-value	ρ	p-value	ρ	p-value	ρ	p-value
BMI	0.017	0.874	0.010	0.931	0.351	0.004	0.342	0.005
SMI	0.376	0.0004	0.374	0.0004	0.448	0.0002	0.438	0.0003
WBPhA	0.134	0.257	0.130	0.275	-0.03	0.831	-0.13	0.353
Fat mass	-0.05	0.649	-0.07	0.496	0.339	0.006	0.299	0.016
BMC	0.508	<.0001	0.486	<.0001	0.512	<.0001	0.524	<.0001
Rt HS	0.290	0.011	0.277	0.015	0.199	0.121	0.088	0.496
Lt HS	0.558	<.0001	0.562	<.0001	0.230	0.073	0.192	0.135
Birth weight	0.197	0.137	0.158	0.238	-0.05	0.733	-0.05	0.696

VC, vital capacity; FVC, forced vital capacity; BMI, body mass index; SMI, skeletal muscle mass index; WBPhA, whole–body phase angle; BMC, bone mineral content; Rt, right; HS, handgrip strength; Lt, left.

ρ is the Spearman's rank correlation coefficient.

Table S3. Univariate correlation analysis of extrapulmonary factors for lung function in all participants

	%VC		%FVC		$\%FEV_1$	
	ρ	*p*-value	P	*p*-value	ρ	*p*-value
BMI	0.2611	0.0012	0.2330	0.0040	0.1195	0.1438
SMI	0.2223	0.0061	0.1686	0.0385	0.1349	0.0987
WBPhA	0.1195	0.1810	0.0415	0.6428	0.0476	0.5954
Fat Mass	0.1014	0.2217	0.0846	0.3082	-0.0458	0.5816
BMC	0.1514	0.0635	0.1048	0.2004	0.1186	0.1470
Rt HS	0.1751	0.0392	0.1007	0.2383	0.1210	0.1560
Lt HS	0.2337	0.0056	0.1789	0.0351	0.1666	0.0500
Birth weight	-0.0567	0.5492	-0.0850	0.3686	-0.0057	0.9524

%, predicted; VC, vital capacity; FVC, forced vital capacity; FEV_1, forced expiratory volume in one second; BMI, body mass index; SMI, skeletal muscle mass index; WBPhA, whole-body phase angle; BMC, bone mineral content; Rt, right; HS, handgrip strength; Lt, left.

ρ is the Spearman's rank correlation coefficient.
